# Intestinal mucoadhesive devices for oral delivery of insulin

**DOI:** 10.1002/btm2.10015

**Published:** 2016-08-19

**Authors:** Amrita Banerjee, JooHee Lee, Samir Mitragotri

**Affiliations:** ^1^ Dept. of Chemical Engineering University of California Santa Barbara Santa Barbara CA 93106; ^2^ Center for Bioengineering University of California Santa Barbara Santa Barbara California 93106

**Keywords:** diabetes, intestinal mucoadhesive patches, oral insulin delivery, permeation enhancers

## Abstract

Oral delivery of proteins such as insulin has been a long‐lasting challenge owing to gastrointestinal degradation and poor permeability of therapeutic macromolecules across biological membranes. We have developed mucoadhesive intestinal devices for oral delivery of insulin to address this challenge. Here we demonstrate a combination of intestinal devices and a permeation enhancer, dimethyl palmitoyl ammonio propanesulfonate (PPS), for oral delivery of insulin. The devices were delivered from a capsule coated with a pH‐responsive enteric coating. The devices adhere to intestinal mucosa, release their protein load unidirectionally, and prevent enzymatic degradation in the gut. Devices were found to completely release their drug load within 3–4 hr and showed excellent strength of mucoadhesion to porcine intestine. Devices loaded with insulin and PPS significantly decreased blood glucose levels by 30 and 33% in diabetic and nondiabetic rats, respectively. These studies demonstrate that intestinal mucoadhesive devices are a promising oral alternative to insulin injections and therefore should be further explored for the treatment of diabetes.

## 1 Introduction

The current trend in drug development is garnered toward peptide/protein‐based therapeutics with approximately 50% of pipeline drugs in the pharmaceutical industry comprising peptide/protein drugs.^1^ This owes to the capability of these macromolecules to bind specifically to their target sites, preventing nonspecific interactions, and thus improving treatment efficacy.^1^ However, peptide/protein‐based drugs face several challenges with respect to oral delivery due to their instability in the gastrointestinal tract (GIT) and poor permeability across biological membranes, which necessitate their parenteral administration.^2^, ^3^ This poses severe limitation for frequently administered drugs, for example insulin, where noncompliance with injections results in inefficient management of diabetes and long‐term complications.^4^, ^5^

To this end, many efforts have been made to develop novel oral delivery technologies for effective administration of therapeutic proteins/peptides.^6^, ^7^, ^8^, ^9^, ^10^ Significant emphasis has been placed on using nanoparticles for delivery of insulin.^11^, ^12^, ^13^, ^14^, ^15^, ^16^ Our laboratory has focused on developing mucoadhesive intestinal devices for oral delivery of therapeutic proteins such as salmon calcitonin, exenatide, and insulin.^17^, ^18^, ^19^, ^20^ Mucoadhesive devices are made from a mixture of mucoadhesive polymers and placed in enteric‐coated capsules for delivery to the small intestine. When taken orally, the capsules dissolve in the intestine and release the devices, which subsequently adhere to the mucous layer of the intestine, swell, and release their drug load over time through dissolution of the device matrix. The devices are coated on all but one side with a water impermeable backing layer to allow unidirectional release of drug. These devices not only help evade the acidic environment of the stomach but also prevent access of proteolytic enzymes present in the GIT to the drug load, therefore prevent enzymatic degradation of therapeutic proteins. In addition, the devices create a high concentration gradient for drug transport, which facilitate uptake of loaded proteins through the intestinal membrane. To further improve transport of drugs across the intestine, the devices can also be loaded with permeation enhancers that can reversibly alter the intestinal absorption barrier and enable higher uptake of drugs.^21^ Previously, we demonstrated that a permeation enhancer PPS (dimethyl palmitoyl ammonio propanesulfonate), enhanced macromolecule absorption after intestinal administration of PPS.^21^ PPS is a zwitterionic surfactant that functions through membrane solubilization and temporarily modulates the intercellular tight junctions thereby promoting paracellular uptake.^21^, ^22^, ^23^ Here, we demonstrate that devices simultaneously loaded with insulin and PPS can be administered orally and possess efficacy in lowering blood glucose levels in both normal and diabetic animals.

## 2 Experimental Section

### 2.1 Materials

Polymers such as Eudragit^®^ E PO and Eudragit^®^ L100 were kind gifts from Evonik Industries (Parsipanny, NJ, USA). Other polymers and drugs such as pectin, carbopol, sodium carboxymethyl cellulose (SCMC), PPS, and ethyl cellulose were purchased from Sigma‐Aldrich (St. Louis, MO, USA). Metoclopramide hydrochloride, Streptozotocin (STZ), sodium citrate buffer pH 4.5, and phosphate buffered saline (PBS) tablets were obtained from Fisher Scientific (Pittsburgh, PA, USA). Devices were pressed using an evacuable pellet press (Pike Technologies, Fitchburg, WI, USA) mounted on a bench top press (Carver, Inc., Wabash, IN, USA). The devices were cut into 2–5‐mm‐sized devices using disposable biopsy punches obtained from Fisher Scientific (Pittsburgh, PA, USA). Bovine serum albumin (BSA), lysozyme, PPS, Fluorescein isothiocyanate (FITC)‐insulin, and human insulin were purchased from Sigma‐Aldrich (St. Louis, MO, USA) while micro bicinchoninic acid (BCA) assay kit was obtained from Life Technologies (Grand Island, NY, USA). Porcine intestine was purchased from Lampire Biologicals Inc (Pipersville, PA, USA). Size 9 capsules and oral capsule dosing kit were bought from Torpac, Inc (Fairfield, NJ, USA). Aimstrip plus blood glucose meter and strips were purchased from Fisher Scientific (Pittsburgh, PA, USA). All animals (6‐weeks‐old male Wistar rats) were purchased from Charles River Laboratories (Wilmington, MA, USA).

### 2.2 Methods

#### 2.2.1 Device preparation

The devices were prepared by mixing three polymers, Eudragit^®^ E PO, pectin, and SCMC in a dry weight ratio of 1:1:2. Precise amount of proteins/enhancers (BSA/lysozyme/insulin/PPS) were added to the mixture to obtain specific % wt/wt loading per device. The mixture was then pressed using a pellet press at 3‐tons pressure into 13 mm discs (∼400 μm thick) and coated three times on one surface and the edges with 5% wt/vol ethyl cellulose in acetone. The 13 mm patches were then cut into either 5 mm discs for all *in vitro* characterization studies or into 2 mm discs for *in vivo* studies using corresponding biopsy punches. For *in vivo* experiments, the devices were placed in Size 9 capsules that were enteric coated with 12.5% wt/vol Eudragit^®^ L100 in isopropanol.

#### 2.2.2 *In vitro* drug release studies

##### 2.2.2.1 Protein release study

For protein release study, BSA and lysozyme were used as model proteins. They were loaded in 5 mm devices (∼17 mg weight) at 10% device wt/wt concentration (∼1.7 mg) and placed in tubes containing 10 ml PBS (pH 7.4). The tubes were subsequently placed on a shaker at 37°C during the entire study period of 5 hr to mimic intestinal conditions including peristaltic motion. At various time intervals, predetermined volumes of solutions from the tubes were removed and replaced with equal volumes of PBS at every point. Protein concentrations (BSA and lysozyme) at each time points were evaluated using micro BCA assay as per manufacture instructions and the absorbance of the samples were determined at 562 nm using a Tecan Infinite M200 Pro plate reader (Tecan US, Inc., Morrrisville, NC, USA). Drug release was analyzed as zero‐order kinetics as described by Gupta et al.^17^ and the percentage cumulative release of protein over time was calculated and plotted as concentration‐time curve profile.

##### 2.2.2.2 Insulin release study

The release of insulin from devices was also conducted using 5‐mm‐sized devices as previously described. The devices were loaded with .1% device wt/wt FITC‐insulin (∼.017 mg). FITC‐insulin concentrations in the samples were determined by measuring the fluorescence of the samples at 494 nm excitation wavelength and 518 nm emission wavelength using Tecan platereader. The release of FITC‐insulin over 5 hr was plotted as percent cumulative amount released over time. Insulin release study in low pH was not conducted since patches are not released from enterically coated capsules in acidic environment such as in the stomach.

##### 2.2.2.3 PPS release study

Five‐millimeter‐sized devices containing .1% wt/wt PPS (∼.017 mg) were prepared and release study was conducted similar to aforementioned protein release study procedure. The concentration of PPS in the samples was evaluated using liquid chromatography–mass spectrometry (LC–MS). For chromatographic separation, Waters alliance 2695 separation module (Waters Corporation, Milford, MA, USA) was used that was attached to Thermo biobasic 18; 150 × 2.1 mm; 5 u pore size column. Mobile phase used for separation was water (A) and methanol (B) containing .1% vol/vol formic acid. The samples were separated using a gradient elution of 5–98% B in the first 15 min, that was kept constant for the next 10 min and brought back to 5% B for a total run period of 30 min. Flow rate used in the study was .2 ml/min and 25 μl was injected into LC using an autosampler. For the MS analysis, Waters Micromass QTOF 2 (Waters Incorporation, Milford, MA, USA) using electrospray ionization (3.5 kV ionization) and Masslinx software was used. PPS concentration was determined from the area under the curve obtained from LC separation and corresponding ion current intensity obtained from MS analysis and based on this information, the release profile of PPS was determined and plotted as percent cumulative PPS release over time.

#### 2.2.3 Mucoadhesion study

The strength of mucoadhesion between devices and intestinal mucosa was evaluated using porcine intestine and as described by Gupta et al.^17^ Briefly, 5 × 5 cm porcine intestine pieces were put in a petri dish containing pH 7.4 PBS. 5‐mm‐sized devices were gently placed on the inner mucosa of the intestine such that their backing layers faced away from the intestinal surface and the whole unit was rocked gently at 37°C for 30 min. Thereafter, the petri dish was placed inside a microbalance containing a cylindrical tube (2 cm length and 1 cm diameter), which was hung inside the balance with a string that passed over a pulley. To the free end of the tube, a drop of acrylate glue was added. The tube was allowed to stick gently to the device and the initial weight of the system with the device attached to the intestine was noted. The tube was then slowly pulled away from the intestine and the weight at the point when the device detached from the mucosa was noted. The differences in these readings were used to evaluate the force of mucoadhesion between the devices and intestinal mucosa.

#### 2.2.4 *In vivo* efficacy studies

The animal experiments were performed according to the University of California Santa Barbara animal care committee guidelines and to the Guide for the Care and Use of Animals of the Institute of Laboratory Animal Resources, National Research Council.

##### 2.2.4.1 Efficacy studies in nondiabetic rats

Efficacy studies in normal rats were conducted in 250–350 g male Wistar rats. The animals were divided into four groups of six animals each and a fifth group containing three animals. Prior to the day of experiment, the animals were fasted overnight but given free access to water. On the day of the experiment, the rats were subcutaneously injected with 5 mg/kg metoclopramide hydrochloride to induce gastric emptying, followed by immediate oral administration of capsules containing either empty devices/insulin devices/insulin‐PPS devices or insulin devices with externally present PPS. The fifth group was injected with 1 U/kg insulin subcutaneously. Insulin dose in all oral administrations was 50 U/kg, while PPS dose in device was 10% device wt/wt (∼.6 mg PPS/animal) and that in capsule was 5 mg. Following capsule dosing, the rats were administered with .5 ml saline solution orally. Compared to oral dose, a much lower parenteral dose was used due to the inherent difference in the bioavailability of proteins administered through parenteral and oral routes. While 100% dose is bioavailable when proteins are administered parenterally, only a fraction enters systemic circulation when given orally. Our devices were developed to improve stability and permeability of orally administered insulin. However, bearing in mind unavoidable drug loss through mucosal bound proteolytic enzymes and insulin's unfavorable physico‐chemical properties such as large size and low lipophilicity, a higher oral dose was utilized for the study compared to injectable dose.^2^, ^24^ Blood glucose levels from the tail vein were measured at different time intervals for a total period of 8 hr using a commercial blood glucose meter.

##### 2.2.4.2 Induction of diabetes in rats

Male Wistar rats weighing 250–350 g were used for the induction of diabetes. The animals were fasted overnight but given free access to water. The basal fasting glucose levels were determined from the tail vein using a commercial glucose meter. The animals were then injected intraperitoneally with 55 mg/kg STZ prepared in .1 mM citrate buffer, pH 4.5 and their fasting blood glucose levels were subsequently analyzed for a 2‐week period after STZ administration. Animals were deemed to be diabetic and used for *in vivo* efficacy studies when their fasting blood glucose values were above 200 mg/dl.

##### 2.2.4.3 Efficacy studies in diabetic rats

After induction of diabetes, the animals were fasted overnight but given free access to water and divided into six groups containing six animals each and a seventh group containing three animals. The first six groups were orally administered with insulin devices, insulin devices containing 10% device wt/wt PPS (∼.6 mg PPS/animal), insulin devices with externally present 5 mg PPS in capsule, empty devices, and insulin‐PPS (5 mg) solution. The seventh group was administered with insulin subcutaneously. Insulin dose used for the study was 100 and 1 U/kg for oral and parenteral administrations, respectively. All animals were injected with 5 mg/kg metoclopramide hydrochloride, prior to the start of treatment, to induce gastric emptying and enable the orally administered capsules to transit from stomach to the intestine. Blood glucose levels from the tail vein were evaluated at different time points ranging from 0 to 8 hr after administration using a commercial blood glucose meter.

### 2.3 Statistical analyses

All data are represented as mean ± standard error (SE). For statistical analyses, Student's T‐Test and/or one‐way ANOVA followed by appropriate post hoc analysis (Graphpad, Prism 6.0, GraphPad Software, La Jolla, CA, USA) was used and a value of *p* < .05 was considered statistically significant.

## 3 Results

**Figure 1:**
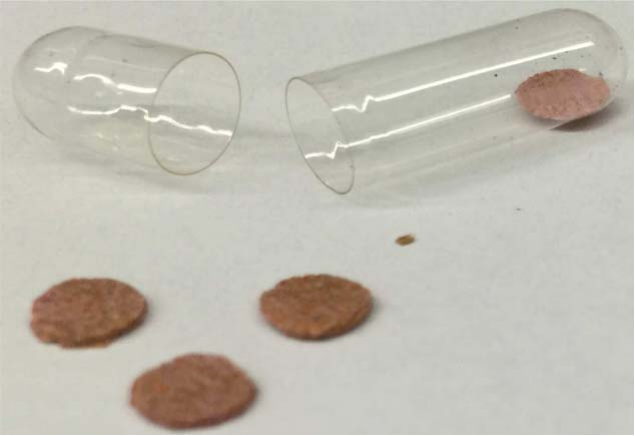
An oral device comprising mucoadhesive patches and enterically coated capsule

Devices were prepared as previously described. Mucoadhesive patches were placed inside enterically coated capsules for site‐specific delivery to the intestine (Figure 1).

### 3.1 Release studies

Release studies were conducted to determine the release profile of different drugs incorporated in the mucoadhesive devices. Ideally, the device must release its content, preferably completely, over time. To assess this, the intestinal mucoadhesive devices made from a mixture of Eudragit® E PO, pectin, and SCMC were loaded with different drugs and the release profiles of the drugs were evaluated.

#### 3.1.1 Protein release

**Figure 2:**
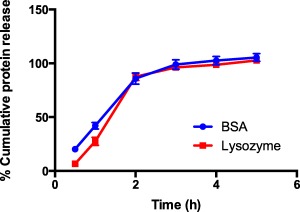
Protein release profile from mucoadhesive devices. Percent cumulative release of bovine serum albumin (BSA) and lysozyme (∼1.7 mg) from mucoadhesive devices (17 mg). Drug release was tested in pH 7.4 PBS at 37°C. Blue closed circles represent BSA release while red closed squares denote lysozyme release profile. Data represented as mean ± SE (*n* = 9)

The release profile of BSA and lysozyme from the devices was evaluated in pH 7.4 PBS. The selection of these proteins was based on the isoelectric point of the proteins such that they display different charge states at pH 7.4. BSA carries a net negative charge while lysozyme carries a net positive charge at pH 7.4. The study was conducted to assess the release profile of both proteins and consequently determine whether charge states of proteins affect their release from the devices. Results obtained from the study demonstrated rapid release of loaded proteins within the first 3 hr followed by a plateau to reach 100% protein release in the next 2 hr (Figure 2). The percentage cumulative release of BSA from the devices was observed to reach 98.9 ± 4.4 within 3 hr of the study and plateaued thereafter for a complete release in the next 2 hr of study. Lysozyme also showed similar release profile and the percentage cumulative release within 3 hr was found to be 96.2 ± 2.3% which reached 100% within the next 2 hr of the study. The concentration‐time curve representing drug release profile between BSA and lysozyme was very similar, indicating that these devices release their load completely regardless of protein charge state.

#### 3.1.2 Insulin release

**Figure 3:**
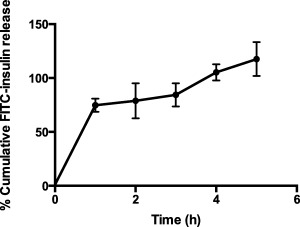
Insulin release profile from mucoadhesive devices. Percent cumulative release of FITC insulin (∼.017 mg) from mucoadhesive devices (17 mg). Drug release was tested in pH 7.4 PBS at 37°C. Data represented as mean ± SE (*n* = 4)

FITC‐insulin was loaded in the devices and its release in pH 7.4 PBS at 37°C was determined over time. The drug was rapidly released from the devices in the first hour of the study to approximately 75% total dose, followed by a slower release over the next 3 hr to reach 100% drug release by 4 hr (Figure 3).

#### 3.1.3 PPS release

**Figure 4:**
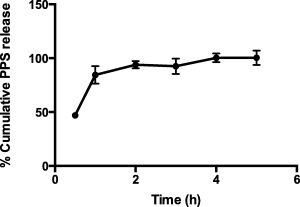
PPS release profile from mucoadhesive devices. Percent cumulative release of PPS (∼.017 mg) from mucoadhesive devices (17 mg). Drug release was tested in pH 7.4 PBS at 37°C. Data represented as mean ± SE (*n* = 3)

The release profile of the intestinal permeation enhancer, PPS was also tested from the devices and found to be similar to that observed with proteins. PPS released rapidly within the first 3 hr followed by a plateau to completely release its drug load within the next 2 hr. The percentage cumulative release was found to reach 92.6 ± 7.2 by 3 hr and subsequently reach 100.3 ± 3.9 by 4 hr (Figure 4). The complete release profile of PPS from these devices suggested that the permeation enhancer, PPS, could be incorporated into insulin devices that would further improve the efficacy of the formulation by facilitating higher uptake of insulin across the intestine.

### 3.2 Mucoadhesion study

**Figure 5:**
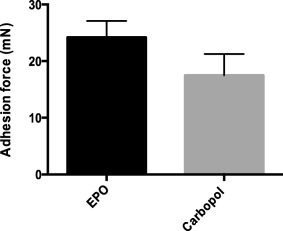
Strength of mucoadhesion of various devices. Adhesion force between E PO/carbopol devices and porcine intestine was measured. Mucoadhesive devices were incubated with porcine intestine for 30 min in pH 7.4 PBS at 37°C and strength of mucoadhesion was measured as the force required to completely detach the devices from the intestine. E PO devices demonstrated significantly higher mucoadhesive force compared to carbopol devices (*p* < .05). Data represented as mean ± SE (*n* = 9)

Mucoadhesion is an essential feature of intestinal devices. Strong mucoadhesive characteristics of the devices enable them to adhere easily to the intestinal mucosa after release from capsules and prevent their dislodging during intestinal peristalsis and passage of food. This study measured the force of mucoadhesion between E PO/carbopol derived devices and intestinal mucosa after 30 min of incubation with porcine intestine. The force of adhesion between E PO device and pig intestine was determined to be 24.2 ± .95 mN, and was significantly higher than that observed between carbopol devices and intestine, found to be 17.5 ± 1.3 mN (Figure 5). In this study, carbopol devices were used as a control and compared with E PO devices since carbopol is also a good mucoadhesive polymer and is widely used in many mucoadhesive formulations for oral/buccal delivery of drugs.^25^ Given that the weight of 5 mm devices is approximately 17 mg (∼.16 mN), the results from this study suggest that E PO devices can endure a force of ∼150 times its own weight. This feature is especially important during transit of food through the intestine where strong mucoadhesive forces between the devices and mucosa will thwart dislocation of the devices once they have adhered to the intestine.

### 3.3 *In vivo* efficacy studies

The purpose of the study was to determine the effectiveness of insulin loaded mucoadhesive devices in the treatment of diabetes through assessment of blood glucose lowering effect in both normal and diabetic rats. The efficacy of the devices was compared to other oral insulin formulation controls as well as the present standard of care, which is subcutaneously injected insulin.

#### 3.3.1 Efficacy studies in nondiabetic rats

**Figure 6:**
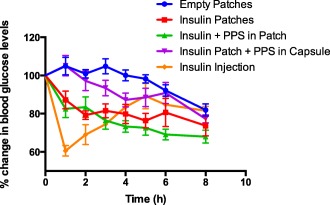
Efficacy of oral insulin formulations in lowering blood glucose levels in nondiabetic rats. Percent reduction in blood glucose levels with time after administration of various formulations in nondiabetic rats. Animals were fasted prior to and during the 8 hr period of study. Oral administration of empty patches (0 U/kg insulin, blue closed circles); insulin patches (50 U/kg insulin, red closed squares); insulin and PPS patches (50 U/kg insulin and ∼.6 mg PPS/animal, green closed triangles); insulin patch and PPS externally placed in capsules (50 U/kg insulin and 5 mg PPS/animal, magenta closed inverted triangles); and subcutaneous insulin injection (1 U/kg insulin, orange closed rhombus) were performed. Blood from tail vein was analyzed for glucose content for 8 hr and data are plotted as percent reduction in blood glucose as compared to baseline (0 hr) glucose levels. Data represented as mean ± SE (*n* = 6). Insulin and Insulin‐PPS devices showed statistically significant difference compared to all other oral control groups (*p* < .05)

Nondiabetic rats were treated with four different oral formulations and their efficacy in lowering blood glucose levels was compared with insulin injection. Rats treated with empty devices showed no significant drop in blood glucose levels for up to 6 hr (92.2 ± 3%) but with an additional 2 hr of fasting, the blood glucose levels dropped by approximately 20% of initial levels (81.9 ± 3.2%) at 8 hr (Figure 6). On the contrary, animals that were orally administered with insulin devices showed an immediate significant drop in blood glucose levels of approximately 13% of initial levels at 1 hr (87.2 ± 4.6%) that decreased further by around 27% by the end of 8 hr (73.7 ± 5.3%). Conversely, rats that were administered with insulin devices along with externally placed PPS in capsules showed a slight increase in blood glucose levels at 1 hr (105.2 ± 5.4%) and only decreased by 10% by 6 hr of study (90.9 ± 5.6%). This group however showed a significant drop by the end of 8 hr of study to 77.3 ± 2.1% of initial levels. The group treated with insulin devices with 10% wt/wt PPS in the device demonstrated best efficacy among the other orally administered formulations. The blood glucose levels dropped significantly by 18% to 82.5 ± 4.6% of initial levels within 1 hr of oral administration and further decreased to 67 ± 3.3% by 8 hr. Parenteral administration of insulin resulted in a significant drop to 60.6 ± 2.7% by 1 hr but increased to 81.5 ± 2.3% by the end of the study period. Overall, a significant difference in blood glucose lowering efficacy (*p* < .05) was found between insulin and insulin‐PPS devices when compared to other oral formulation controls.

#### 3.3.2 Efficacy studies in diabetic rats

**Figure 7:**
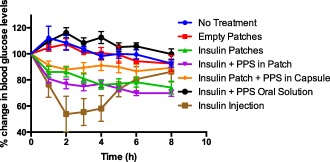
Efficacy of oral insulin formulations in lowering blood glucose levels in diabetic rats. Percent reduction in blood glucose levels with time after administration of various formulations in diabetic rats. Animals were fasted prior to and during the 8 hr period of study. Animals were either given no treatment (blue closed circles) or administered orally with empty patches (0 U/kg insulin, closed red squares); insulin patches (100 U/kg insulin, green closed triangles); insulin and PPS patches (100 U/kg insulin and ∼.6 mg PPS/animal, magenta closed inverted triangles); insulin patch and PPS externally placed in capsules (100 U/kg insulin and 5 mg PPS/animal, orange closed rhombuses); solution of insulin and PPS orally (100 U/kg insulin and 5 mg PPS, black closed circles); and subcutaneous insulin injection (1 U/kg insulin, closed brown squares). Blood from tail vein was analyzed for glucose content for 8 hr and data are plotted as percent reduction in blood glucose as compared to baseline (0 hr) glucose levels. Data represented as mean ± SE (*n* = 6). Insulin and Insulin‐PPS devices showed statistically significant difference compared to all other oral control groups (*p* < .05)

The efficacy in lowering blood glucose levels of six different formulations was tested in STZ induced diabetic rats and compared with no treatment control. Rats that were not treated with any formulation besides metoclopramide injection at the beginning of the study showed an increase in blood glucose levels during the first 3 hr of the study and did not show any decrease in blood glucose levels for up to 6 hr (99.5 ± 3.6%) (Figure 7). At the end of the study period at 8 hr, the blood glucose levels decreased by approximately 7% to 92.6 ± 2.7%. The group treated with empty devices also showed an initial increase in blood glucose levels and remained at baseline levels till 5 hr (99.3 ± 6.4%) and decreased only by 8% by 8 hr to 92.5 ± 6.3%. In contrast, the group treated with insulin devices demonstrated a significant drop in blood glucose level to 86.2 ± 4.3% by 1 hr that kept decreasing to greater than 25% to 74.2 ± 4.9% by the end of the study period. The group treated with insulin devices and PPS externally placed in capsule also showed a decrease in blood glucose levels with time but not as efficiently as insulin device group. The decrease plateaued to around 10% of initial levels between 2 and 8 hr with a total drop to 89.3 ± 3.8% at 8 hr. The insulin and PPS in device treatment group demonstrated greatest efficacy in blood glucose lowering as was observed with nondiabetic rats. The blood glucose levels dropped significantly by around 20% to 81 ± 3.2% by 1 hr and progressively decreased to 69.9 ± 2.4% by 8 hr. The blood glucose lowering effect of orally administered insulin and PPS solution was also tested and it did not show a drop in blood glucose level by the end of the study period and remained at 99.8 ± 4% at 8 hr. Animals treated with insulin injection showed a drop in blood glucose levels at 1 hr to 76.5 ± 9.9% that decreased further to 53–58% of initial levels between 2 and 4 hr, after which it progressively increased to 86.2 ± 7.9% by 8 hr. As observed in the study on nondiabetic rats, here too the insulin and insulin‐PPS device groups demonstrated statistically significant difference in blood glucose lowering (*p* < .05) when compared to other oral formulation controls. The delay between insulin release from patches and maximum *in vivo* efficacy can be attributed to the time required for insulin to traverse through the intestine and appear in the blood stream. Thereafter the lag time for biological response adds to the interval between insulin release and efficacy, which is evident from insulin injection groups where maximal efficacy is observed after 1–2 hr of drug administration.

## 4 Discussion

**Figure 8:**
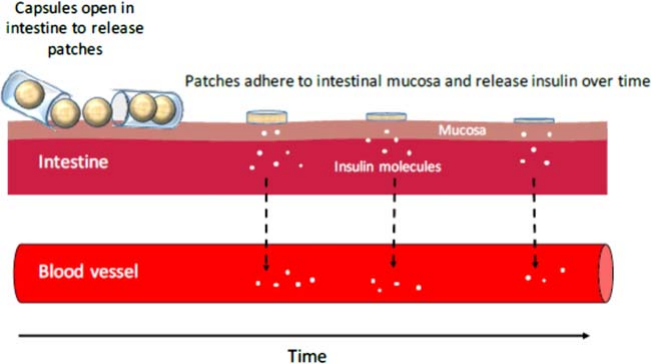
Schematic representation of mode of action of oral devices. Mucoadhesive patches are released from enterically coated capsules in the intestine where it adheres to the mucosa and releases insulin over time

Oral delivery of therapeutic proteins faces several problems that necessitate administration of therapeutic proteins/peptides through parenteral routes. For diseases that require occasional administration of therapeutic proteins, this may be acceptable to patients, but for chronic diseases such as diabetes that require frequent administration of insulin, the pain of injections leads to poor adherence to treatment, suboptimal therapy and increased hospitalization/morbidity.^4^ A study by Morris et al. found that around 28% of population with Type 1 diabetes mellitus did not adhere to prescribed insulin injection dose.^5^ Oral delivery has the potential to address this limitation. However, proteins delivered orally are easily degraded by the enzymes and the acid in the gut. In addition, they have poor GI permeability that limits their oral absorption. Insignificant amounts of insulin are found to enter systemic circulation after oral administration under native conditions.^26^ Our devices release drug loaded patches at the intestine and enhance oral bioavailability by providing high‐concentration, protected drug depots on the surface of the intestine (Figure 8). To further improve oral absorption, the devices can be loaded with permeation enhancers such as PPS that work by temporarily opening the tight junctions in the intestine.

The devices completely released their content, irrespective of the charge of the cargo. Eudragit^®^ E PO (polycationic type E polymer) is widely used as a protective coating polymer and is known to swell and become permeable above pH 5.0 to enable controlled release.^27^ Pectin is a natural polysaccharide widely used in food and pharmaceutical industry and has applications in oral drug delivery due to its mucoadhesive and gelling properties.^28^, ^29^ Sodium carboxy methylcellulose, conversely, is a water‐soluble mucoadhesive polymer that is used in oral delivery because of its property to form a swellable matrix at the pH of the intestine for controlled drug release.^6^, ^10^, ^30^ The swelling and gel forming properties of the polymers used in the device may have together contributed to complete release of loaded drugs from its matrix.

The E PO devices showed excellent mucoadhesive strength for interactions with porcine intestine. Glycoproteins in the mucous layer carry a net negative charge, which make the mucous surface possess an overall negative charge.^31^ We postulate that Eudragit^®^ E PO being a cationic polymer can therefore interact strongly with the negatively charged mucous layer. Conversely, pectin and SCMC, despite being anionic polymers form hydrogen bonds with mucin and atomic force microscopy studies have revealed that the electrostatic charge based repulsion between pectin and mucin molecules causes uncoiling of polymer chains, entanglement and formation of bonds for adsorption.^32^, ^33^

The efficacy of the devices was also validated *in vivo* in both nondiabetic and diabetic rats. Devices loaded with insulin or insulin‐PPS demonstrated significant reduction in blood glucose levels compared to formulation controls with an approximate 30% decrease in glucose levels in both normal and diabetic rats. Administration of the insulin device formulations led to an immediate drop in blood glucose levels, which was sustained for up to 8 hr especially in diabetic animals. The efficacy of the devices was further improved by the co‐administration of insulin and PPS, which functions as a permeation enhancer and promotes paracellular transport of drugs across intestinal barriers.

Other mucoadhesive polymer based devices have also been developed for oral delivery of proteins. Recently, Lee et al. developed dual sided Janus devices where one side strongly adhered to gastrointestinal mucous layer while the other repelled food and liquids to prevent dislodgement during passage of food for longer GI residence.^34^ Earlier, a four layered gastrointestinal mucoadhesive patch systems (GI‐MAPS) were developed for oral delivery of granulocyte colony‐stimulating factor protein.^35^ Apart from the oral route, various mucoadhesive tablets, patches, films, gels, and ointments have been developed for drug delivery through buccal, nasal, ocular, vaginal, and rectal routes.^30^, ^36^

In addition to the aforementioned formulations, many nanoparticle‐based oral formulations of insulin using both targeted and nontargeted systems have been studied and have shown similar efficacy in decreasing blood glucose levels as observed with our devices. Jin et al. prepared insulin loaded nanoparticles using trimethyl chitosan attached to a targeting peptide and demonstrated approximately 28% reduction in blood glucose levels at 50 U/kg insulin dose.^16^ Another chitosan nanoparticle based oral insulin formulation made by Mukhopadhyay et al. showed similar *in vivo* efficacy of around 29% decrease in blood glucose levels using 50 U/kg dose.^37^ A few other chitosan based insulin nanoparticles such as chitosan‐dextran sulfate nanoparticles prepared by Sarmento et al. demonstrated a 35% reduction in blood glucose levels^38^ while those made using lauryl chitosan sulfate by Rekha and Sharma showed a 34% decrease.^38^, ^39^ Zhang et al. tested Eudragit^®^ L100‐cysteine nanoparticles *in vivo* and found similar efficacy of 28% reduction in blood glucose levels at 50 U/kg insulin dose.^40^ In addition to chitosan and Eudragit^®^ derived nanoparticles, researchers have also studied PLGA (poly[lactic‐co‐glycolic acid]) nanoparticles with or without targeting ligands or cell penetrating peptides for oral delivery of insulin and observed blood glucose lowering efficacy ranging between 30 and 50%.^41^, ^42^, ^43^ Based on these examples and others, although it is evident that nanoparticles demonstrate good efficacy in antidiabetes therapy, they often involve complex procedures for synthesis leading to difficulty in scale‐up, suffer from dose dilution in the gut and have unknown long‐term safety profile especially for chronic treatment such as that for diabetes where biodegraded products of polymers may accrue in the cells.^15^, ^44^ Mucoadhesive devices are easy to prepare and minimize dose dilution due to the presence of backing layer on the devices while creating a high concentration depot for drug absorption. The device polymer such as E PO does not dissolve at intestinal pH to be absorbed and pectin being a naturally occurring polysaccharide has a good safety profile.^45^ The release of insulin from the patches could be influenced by the highly variable intestinal environment that changes with fasting and fed conditions and should be taken into consideration for future *in vitro* characterization of patches. In addition, the efficacy of the devices could be potentially further enhanced by incorporation of additional enhancing strategies such as cell penetrating peptides or vitamin B12 as targeting ligands.^11^, ^41^ Future studies should focus to explore these possibilities.

## 5 Conclusions

A novel oral intestinal mucoadhesive device formulation of insulin and permeation enhancer, PPS has been developed and characterized. Drug load from the devices was completely released and the devices demonstrated good mucoadhesive strength that could withstand ∼150 times their own weight as well as significant blood glucose lowering efficacy *in vivo*. Oral administration of insulin using intestinal mucoadhesive devices avoids the need for routine insulin injections for the management of diabetes. Taken together, the study suggests that intestinal mucoadhesive devices provide an effective alternative to insulin injections for management of diabetes that can significantly improve the quality of life of patients suffering from this chronic disease.

## Abbreviations

ANOVA: analysis of variance
BCA: bicinchoninic acid
BSA: bovine serum albumin
GIT: gastrointestinal tract
FITC: fluorescein isothiocyanate
LC–MS: liquid chromatography–mass spectrometry
PBS: phosphate buffered saline
PPS: dimethyl palmitoyl ammonio propanesulfonate
SCMC: sodium carboxy methyl cellulose
SE: standard error
STZ: streptozotocin
